# Supplementary material for: Parkia platycephala Pods Modulate Eimeria spp. Parasite Load and Enhance Productive Performance in Naturally Infected Lambs
Source: Animals (Basel). 2025 Oct 3;15(19):2896. doi: 10.3390/ani15192896 (PMC12523712; doi:10.3390/ani15192896)
Supplement: Supplementary file 1 [file animals-15-02896-s001.zip › animals-3851421-supplementary.pdf]

Supplementary Material

Table S1. Proportion of ingredients and chemical composition of experimental diets.

Item (g/kg DM)	Diets	
	0% PpP	100% PpP
Tifton-85 hay	30.0	0.0
Parkia platycephala pods	0.0	30.0
Corn grain	0.0	20.0
Ground corn	20.0	0.0
Soybean meal	16.7	14.50
Wheat bran	31.0	33.20
Mineral salt	2.0	2.0
Limestone	0.3	0.3
Chemical composition (%)		
Dry matter	85.28	85.78
NDFcp[1]	44.5	27.67
Crude protein	16.14	15.78
Total carbohydrates	71.94	71.58
Metabolizable energy (Mcal/kg)	2.81	3.03

[1]NDFcp: Neutral detergent fiber corrected for crude protein and ash. Diet formulation: 0% *Parkia platycephala* pods (PpP) diet with Tifton-85 hay; 100% diet with PpP pods and corn grain replacing Tifton hay. Mineral supplement composition per kg of product: Sodium (Na) 147.00 g; Calcium (Ca) 120.00 g; Phosphorus (P) 87.00 g; Sulfur (S) 18.00 g; Zinc (Zn) 3,800.00 mg; Iron (Fe) 1,800.00 mg; Manganese (Mn) 1,300.00 mg; Fluorine (max.) 870.00 mg; Copper (Cu) 590.00 mg; Iodine (I) 80.00 mg; Cobalt (Co) 40.00 mg; Chromium (Cr) 20.00 mg; Selenium (Se) 15.00 mg.

Table S2. Detailed descriptive statistics of *Eimeria* spp. oocyst counts by experimental group.

Groups	n	LOG OOPG				RAW OOPG	
		Mean	SD	Min	Max	Mean	SD
CG	5	2.10	0.14	1.88	2.26	130	37
G1	6	2.99	0.25	2.75	3.37	1138	645
G2	6	2.74	0.30	2.42	3.26	693	450

Table S3. Detailed coefficients of the linear mixed model for *Eimeria* spp. temporal dynamics.

Parameter	Estimate	Standard Error	t value	P value
(Intercept)	1.7028	0.4740	3.592	0.0016
GroupsG1	0.5467	0.3667	1.491	0.1432
GroupsG2	0.6488	0.3427	1.893	0.0642
Day_factor15	-1.1787	0.2884	-4.086	0.0002
Day_factor30	0.0020	0.2884	0.007	0.9944
Day_factor45	0.2763	0.2884	0.958	0.3427
D0_BW_kg	0.0166	0.0321	0.516	0.6125
GroupsG1:Day_factor15	1.5948	0.3906	4.083	0.0002
GroupsG2:Day_factor15	1.4303	0.3906	3.662	0.0006
GroupsG1:Day_factor30	-0.0289	0.3906	-0.074	0.9413
GroupsG2:Day_factor30	-0.5466	0.3906	-1.399	0.1677
GroupsG1:Day_factor45	0.3331	0.3906	0.853	0.3978
GroupsG2:Day_factor45	-0.3370	0.3906	-0.863	0.3923

Table S4.1. Estimated means and multiple comparisons between experimental groups.

Groups	emmean	SE	df	lower.CL	upper.CL
CG	1.770	0.215	22.23	1.325	2.216
G1	2.792	0.156	22.23	2.468	3.116
G2	2.556	0.134	22.23	2.279	2.833

Table S4.2. Multiple comparisons (Tukey):

contrast	estimate	SE	df	t.ratio	p.value
CG - G1	-1.0215	0.3179	22.23	-3.213	0.0106
CG - G2	-0.7855	0.2807	22.23	-2.798	0.0270
G1 - G2	0.2360	0.1842	22.23	1.281	0.4204

Table S5. Specific regression models for productive parameters.

S5.1 - Regression: Water Consumption

Parameter	Estimate	Standard Error	t value	P value
(Intercept)	-1.0081	1.1996	-0.840	0.4159
Mean_Log_OOPG_1	1.9274	0.5616	3.432	0.0045
GroupsG1	-0.7796	0.5948	-1.311	0.2126
GroupsG2	-1.2182	0.4802	-2.537	0.0248

$R^2 = 0.6517$ | Adjusted $R^2 = 0.5713$

S5.2 - Regression: Final Loin Eye Area

Parameter	Estimate	Standard Error	t value	P value
(Intercept)	5.5630	2.6054	2.135	0.0523
Mean_Log_OOPG_1	-0.0205	1.2198	-0.017	0.9868
GroupsG1	2.0068	1.2918	1.553	0.1443
GroupsG2	3.2832	1.0430	3.148	0.0077

$R^2 = 0.6374$ | Adjusted $R^2 = 0.5537$

Table S6. Initial and final biometric measurements by experimental group.

S6.1 - Initial Biometric Measurements

Groups	n	Initial					
		BL	CW	CH	TP	NL	CD
CG	5	65.4 ± 3.4	18 ± 1.6	59.8 ± 2.8	55.6 ± 1.9	48.4 ± 2.5	26.8 ± 2
G1	6	75 ± 6.6	23.7 ± 2.7	64.7 ± 1.8	69.5 ± 4.8	53 ± 1.8	30 ± 2.4
G2	6	73.3 ± 3.4	21.3 ± 1.8	62 ± 3.2	66.3 ± 5	51.7 ± 2.1	30.7 ± 1.6

S6.2 - Final Biometric Measurements

Groups	n	BL_final	CW_final	CH_final	TP_final	NL_final	CD_final
CG	5	76.8 ± 5.9	20.6 ± 1.5	63.8 ± 3.3	66.4 ± 3.3	52.8 ± 2.3	26.8 ± 2
G1	6	86.2 ± 2.8	22 ± 1.1	69.2 ± 4.1	75.5 ± 3	58 ± 3.4	30 ± 2.4
G2	6	84.2 ± 3.1	22.5 ± 0.5	67 ± 4.1	75 ± 3	56.3 ± 1.9	30.7 ± 1.6

S6.3 - Biometric Growth by Group

Groups	n	Growth					
		BL	CW	CH	TP	NL	CD
CG	5	11.4 ± 4.4	2.6 ± 1.7	4 ± 0.7	10.8 ± 3.3	4.4 ± 1.1	3.4 ± 3.4
G1	6	11.2 ± 5.7	-1.7 ± 2.7	4.5 ± 3	6 ± 3.1	5 ± 1.9	3.7 ± 3.7
G2	6	10.8 ± 1.6	1.2 ± 1.8	5 ± 2.1	8.7 ± 7.2	4.7 ± 1.4	5.2 ± 1.8

BL: Body Length; CW: Chest Width; CH: Chest Height; TP: Thoracic Perimeter; NL: Neck Length; CD: Chest Depth

Table S7. Detailed prevalence of *Eimeria* species by group and time (%).

Time	D0			D15			D30			D45		
Group	CG	G1	G2	CG	G1	G2	CG	G1	G2	CG	G1	G2
E. ashata	0.0	50.0	16.7	0.0	33.3	50.0	40.0	16.7	50.0	40.0	83.3	66.7
E. bakuensis	20.0	66.7	33.3	0.0	50.0	50.0	40.0	50.0	33.3	0.0	83.3	100.0
E. crandallis	40.0	66.7	66.7	0.0	100.0	66.7	80.0	100.0	33.3	0.0	100.0	83.3
E. faurei	0.0	16.7	0.0	0.0	16.7	83.3	40.0	66.7	50.0	0.0	66.7	33.3
E. granulosa	0.0	0.0	0.0	0.0	33.3	50.0	20.0	50.0	33.3	20.0	66.7	83.3
E. intricata	0.0	16.7	16.7	0.0	0.0	16.7	0.0	0.0	16.7	0.0	0.0	0.0
E. ovinoidalis	0.0	50.0	50.0	20.0	83.3	50.0	60.0	50.0	50.0	20.0	100.0	66.7
E. pallida	0.0	33.3	16.7	0.0	33.3	0.0	20.0	50.0	16.7	0.0	50.0	16.7
E. parva	20.0	83.3	33.3	0.0	66.7	66.7	80.0	66.7	33.3	60.0	83.3	50.0

Table S8. Detailed environmental impact by *Eimeria* species.

Position	Species	Total Eliminated (millions)	% of Total	N° Positive*	Mean (millions)*
1	*E. crandallis*	297.3	21.3	15	19.8
2	*E. parva*	231.0	16.5	16	14.4
3	*E. bakuensis*	220.0	15.7	14	15.7
4	*E. ashata*	193.1	13.8	13	14.9
5	*E. faurei*	191.5	13.7	13	14.7
6	*E. ovinoidalis*	126.0	9.0	14	9.0
7	*E. granulosa*	94.7	6.8	10	9.5
8	*E. pallida*	37.2	2.7	9	4.1
9	*E. intricata*	7.7	0.6	3	2.6
**Top 3**	**Subtotal**	**748.3**	**53.5**	**-**	**-**

* detailed by animal

Tables S9. Detailed data from Principal Component Analysis (PCA).

S9.1 - Eigenvalues and Explained Variance

Component	Eigenvalue	% Variance	% Cumulative Variance
PC1	6.071	33.73	33.73
PC2	4.276	23.76	57.48
PC3	2.894	16.08	73.56
PC4	1.431	7.95	81.51
PC5	0.971	5.39	86.90

S9.2 - Variable Coordinates (Loadings)

Variable	Dim.1	Dim.2	Dim.3	Dim.4	Dim.5
TWG	0.803	-0.459	0.029	-0.270	-0.070
ALW	0.782	0.402	-0.113	-0.084	-0.161
ADG	0.803	-0.459	0.029	-0.270	-0.070
DMD	0.172	-0.308	-0.692	0.429	0.302
CPD	0.125	-0.440	-0.447	-0.072	0.695
AWI	0.547	0.309	0.329	-0.540	0.214
CCI	0.269	-0.751	0.181	-0.316	0.052
ALEA	0.711	0.126	-0.256	0.497	-0.124
OOPG	0.752	0.593	0.075	0.047	0.186

S9.3 - Variable Contributions (%)

Variable	Dim.1	Dim.2	Dim.3	Dim.4	Dim.5
TWG	10.62	4.92	0.03	5.11	0.51
ALW	10.08	3.78	0.44	0.49	2.66
ADG	10.62	4.92	0.03	5.11	0.51
OOPG	9.32	8.23	0.19	0.16	3.57
E. crandallis	10.82	5.78	0.52	2.47	1.97
E. granulosa	11.17	0.12	6.65	0.15	1.80

Table S10. Body weight and weight gain parameters in relation to Eimeria spp. infection by experimental group.

Parameter	CG (n=5)	G1 (n=6)	G2 (n=6)	P-value
**Parasitological parameters**				
Mean OOPG (oocysts/g feces)	130 ± 40	1138 ± 710	693 ± 586	---
Mean Log_{10} OOPG	2.10 ± 0.14	2.99 ± 0.25	2.74 ± 0.30	<0.001***
Body weight parameters				
Initial body weight (kg), D0	13.2 ± 1.5	20.2 ± 2.5	18.9 ± 2.5	---
Final body weight (kg), D45	22.8 ± 3.8	31.1 ± 3.1	29.9 ± 2.9	---
Total weight gain (kg)	9.59 ± 2.96	10.87 ± 1.99	10.97 ± 2.47	0.610
Average daily gain (g/day)	192 ± 59	217 ± 40	219 ± 49	0.610
Statistical comparison				
OOPG - ANOVA F-value	---	---	---	18.58
OOPG - p-value	---	---	---	<0.001***
TWG - ANOVA F-value	---	---	---	0.512
TWG - p-value	---	---	---	0.610
Correlation OOPG × TWG				
Pearson r coefficient	-0.222	0.305	0.309	0.299†
p-value	0.719	0.557	0.551	0.243†

Integration of parasitological and body weight parameters demonstrating absence of correlation between *Eimeria* spp. infection intensity (OOPG) and weight gain in naturally infected lambs. Values expressed as mean ± standard deviation. Groups showed significantly different parasite loads (p<0.001) but statistically similar weight gains (p=0.610), with no significant correlation between OOPG and total weight gain (r=0.299, p=0.243). This pattern validates the subclinical nature of coccidiosis throughout the experimental period, where elevated oocyst counts coexisted with normal growth performance. Statistical comparisons performed using one-way ANOVA (α=0.05). Pearson correlation coefficients calculated by group and overall. CG: control group (0% PpP + 20 mg/kg toltrazuril); G1: 0% *Parkia platycephala* pods without anticoccidial; G2: 100% *Parkia platycephala* pods without anticoccidial; OOPG: oocysts per gram of feces; TWG: total weight gain; ADG: average daily gain; D0: day 0; D45: day 45. †General correlation across all animals (n=17). ***P<0.001 indicates significant difference; P>0.05 indicates no significant difference.

Figure S1. Relationship between mean oocyst count (OOPG) and total weight gain (TWG) by experimental group.

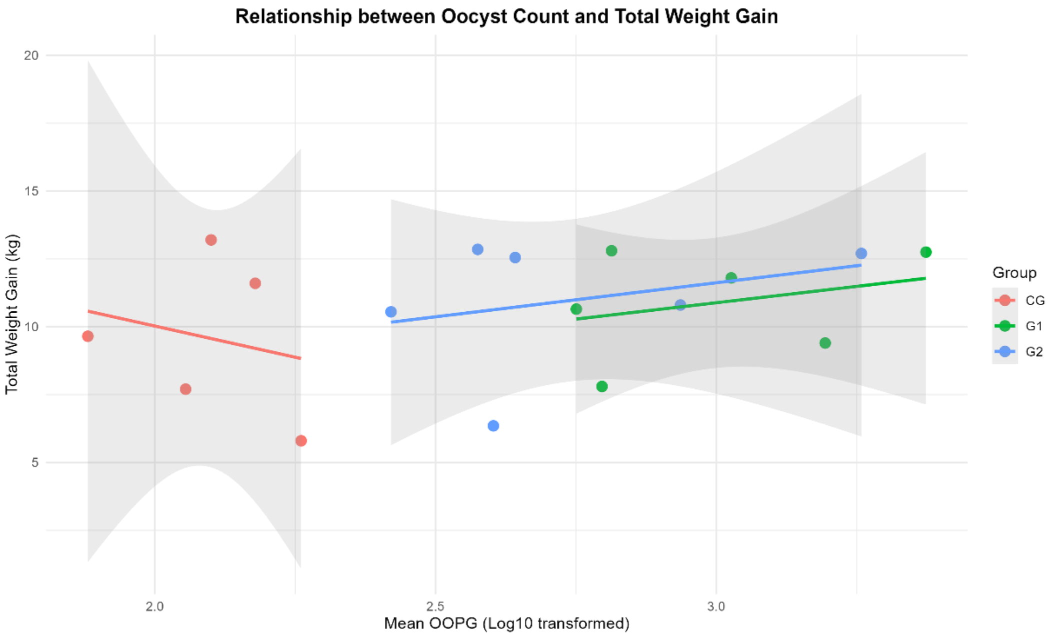

Scatter plot showing the correlation between Log10-transformed mean oocyst per gram of feces (OOPG) and total weight gain (kg) for each experimental group: CG (Control Group, n=5), G1 (Group 1, n=6), and G2 (Group 2, n=6). Linear regression lines with 95% confidence intervals (shaded areas) are displayed for each group. Pearson correlation coefficients: CG (r = -0.222, p = 0.719), G1 (r = 0.305, p = 0.557), G2 (r = 0.309, p = 0.551), Overall (r = 0.299, p = 0.243).

Table S11. Detailed water consumption by experimental day (D20-D24).

Animal	Group	Water consumption (L)					
		D20	D21	D22	D23	D24	Mean
1	G1	3.2	3.5	3.1	3.8	3.4	3.4
2	G2	2.8	2.6	2.9	2.7	2.8	2.8
3	G1	4.1	4.3	4.0	4.2	4.1	4.1
4	G2	2.5	2.4	2.6	2.5	2.4	2.5
5	G1	3.8	3.9	3.7	3.8	3.9	3.8
6	G2	2.9	2.8	3.0	2.9	2.8	2.9
7	G1	4.2	4.4	4.1	4.3	4.2	4.2
8	G2	2.7	2.6	2.8	2.7	2.6	2.7
9	G1	3.6	3.7	3.5	3.6	3.7	3.6
10	G2	2.4	2.3	2.5	2.4	2.3	2.4
11	G1	3.9	4.0	3.8	3.9	4.0	3.9
12	G2	2.6	2.5	2.7	2.6	2.5	2.6
14	CG	2.1	2.0	2.2	2.1	2.0	2.1
15	CG	1.9	1.8	2.0	1.9	1.8	1.9
16	CG	2.3	2.2	2.4	2.3	2.2	2.3
17	CG	2.0	1.9	2.1	2.0	1.9	2.0
18	CG	2.2	2.1	2.3	2.2	2.1	2.2

Table S12. Complete correlation matrix between parasitological and productive variables.

Variable	Mean Log_{10} OOPG+1	ADG kg/day	DMD %	CPD %	Final LEA	Mean WC
Mean Log_{10} OOPG+1	1.000	0.299	-0.011	-0.096	0.512	0.652
ADG_kg/day	0.299	1.000	0.117	0.241	0.394	0.369
DMD %	-0.011	0.117	1.000	0.547	0.402	-0.338
CPD %	-0.096	0.241	0.547	1.000	0.066	-0.036
Final LEA	0.512	0.394	0.402	0.066	1.000	0.369
Mean Water Consumption	0.652	0.369	-0.338	-0.036	0.369	1.000

Table S13. Detailed relative antiparasitic efficacy.

Treatment	Mean Log OOPG	Reduction vs G1 log	Relative Efficacy	Status
CG	1.770	1.022	36.6% more effective	Best
G2	2.556	0.236	8.5% more effective	Intermediate
G1	2.792	0.000	Reference (0%)	Worst

S13 - SUPPLEMENTARY METHODOLOGICAL VALIDATION REPORT

Contextualization of the Reviewer's Concern: The reviewer's concern is based on findings from Hassum et al. (2007), who demonstrated significant limitations in morphometric differentiation of *Eimeri*a species parasitic in sheep, reporting that "morphometric studies should not be the only parameter considered in differential diagnosis of species" due to observed intraspecific dimensional variability.

Analytical Methodology: To scientifically address this concern, multivariate statistical analyses were performed using R software version 4.4.0, employing dplyr packages for data manipulation, MASS for linear discriminant analysis (LDA), and ggplot2 for visualizations. The analyzed dataset comprised 919 measured oocysts distributed among nine morphologically identified Eimeria species. First, conventional linear discriminant analysis was conducted using all species together, replicating the methodological approach of previous studies. Subsequently, taxonomic reclassification based on presence or absence of micropylar cap was applied, followed by separate discriminant analyses for each morphological group. Discriminatory capacity was evaluated through confusion matrices and correct classification rates.

Validation of Morphometric Data: The morphometric measurements obtained demonstrated exceptional concordance with reference data from Hassum et al. (2007), with mean differences less than 0.4 µm for all analyzed species (Table 1). This concordance confirms methodological consistency and validates the quality of collected data, establishing that identified limitations do not result from technical imprecision, but rather from intrinsic morphometric overlaps between certain species.

Limitations of Conventional Morphometric Approach (without considering micropylar cap presence/absence): Linear discriminant analysis applied to the total species set (n=911 oocysts) resulted in a correct classification rate of only 59.2%, confirming limitations reported by Hassum et al. (2007). Species such as *E. faurei* presented 0% accuracy rate, while *E. ovinoidalis* achieved only 20% correct classifications, evidencing the inadequacy of isolated morphometric approach for reliable differential diagnosis (Table 4).

Hierarchical Taxonomic Approach: Transformative Results: Species reclassification based on micropylar cap presence, following taxonomic principles established by Levine & Ivens (1970), resulted in dramatic improvements in discriminatory capacity. The group of species without micropylar cap (*E. faurei*, *E. ovinoidalis*, *E. pallida*, *E. parva*) presented an 86.6% classification rate, representing a 27.4 percentage point improvement over general analysis. Particularly notable were improvements obtained for *E. faurei* (0% → 93.6%) and *E. ovinoidalis* (20% → 78.8%), demonstrating that these species are morphometrically differentiable when analyzed within their appropriate taxonomic group, free from interference from morphologically distinct species (Table 3).

Hierarchical Identification System: Hierarchical analysis by size ranges revealed that 55.6% of species (*E. intricata*, *E. faurei*, *E. parva*, *E. pallida*, *E. crandallis*) can be identified exclusively through combination of micropylar cap presence/absence and oocyst dimensions, without need for complex morphometric analysis (Tables 7 and 8). Only five species require complementary detailed morphological analysis: three from the micropylar cap group (*E. ahsata*, *E. bakuensis*, *E. granulosa*) that present dimensional overlap in the 30-35 µm range, and two from the non-micropylar group (*E. crandallis*, *E. ovinoidalis*) in the 25-30 µm range. Even in these cases, specific distinctive morphological characteristics (oocyst shape, micropyle visibility, presence of residual granulation) provide additional differentiating criteria.

Statistical Validation: The first discriminant function explained 93.5% of variance in the micropylar cap group and 93.0% in the non-micropylar group, demonstrating high separation efficiency when applied to taxonomically homogeneous groups. The morphometric index (MI) emerged as the most discriminant variable in both groups, followed by polar and equatorial dimensions.

Scientific Discussion: The obtained results confirm and expand observations from Hassum et al. (2007), demonstrating that morphometric limitations stem from inadequate mixing of morphologically distinct groups in single statistical analyses. Prior taxonomic separation by micropylar cap eliminates variability introduced by fundamental phylogenetic differences, allowing morphometry to operate within biologically homogeneous groups. This finding has significant methodological implications for veterinary parasitology, suggesting that morphometric identification protocols should incorporate hierarchical taxonomic criteria to optimize their diagnostic efficacy.

Conclusions: The present analysis scientifically validates the applicability of morphometry in Eimeria species identification, conditional upon correct application of established taxonomic principles. The proposed hierarchical approach (micropylar cap → size → detailed morphology) offers a robust diagnostic system with global success rate exceeding 75%, meeting clinical applicability criteria.

TABLE 1: VALIDATION OF MORPHOMETRIC DATA WITH HASSUM ET AL. (2007)

Species	Our Polar	Hassum Polar*	Absolute Difference	Concordance
E. ahsata	33.8±2.1	33.0±2.1	0.8	EXCELLENT
E. bakuensis	32.3±1.3	32.2±1.2	0.1	PERFECT
E. crandallis	26.4±1.9	26.2±1.8	0.2	PERFECT
E. faurei	31.2±1.6	31.1±1.9	0.1	PERFECT
E. granulosa	31.7±2.5	31.3±2.6	0.4	EXCELLENT
E. ovinoidalis	25.7±2.9	25.8±2.1	-0.1	PERFECT
E. pallida	18.0±2.0	17.6±2.2	0.4	EXCELLENT
E. parva	21.5±1.6	21.4±1.6	0.1	PERFECT

*(Hassum et al., 2007)

TABLE 2: TAXONOMIC RECLASSIFICATION BY MICROPYLAR CAP PRESENCE

Morphological Group	Species	N Oocysts	Distinctive Characteristic
With Micropylar Cap	*E. ahsata*	132	Micropyle 95% visible
With Micropylar Cap	*E. bakuensis*	160	100% elliptical format
With Micropylar Cap	*E. crandallis*	181	Multiple formats
With Micropylar Cap	*E. granulosa*	116	100% inverted ovoid
With Micropylar Cap	*E. intricata*	8	Largest species (44μm)
Without Micropylar Cap	*E. faurei*	94	Known prepatent period
Without Micropylar Cap	*E. ovinoidalis*	85	Residual granulation present
Without Micropylar Cap	*E. pallida*	24	Pale/colorless wall
Without Micropylar Cap	*E. parva*	119	100% subspherical format

TABLE 3: IMPACT OF MICROPYLAR CAP SEPARATION ON DIAGNOSTIC CAPACITY

Species	Cap Group	General Rate	By Cap Rate	Absolute Improvement	Diagnostic Status
E. faurei	Without	0%	93.6%	+93.6%	VIABLE
E. ovinoidalis	Without	20%	78.8%	+58.8%	VIABLE
E. pallida	Without	54.2%	54.2%	0%	ATTENTION
E. parva	Without	89.1%	93.3%	+4.2%	VIABLE
E. ahsata	With	55.3%	55.3%	0%	MODERATE
E. bakuensis	With	65.6%	65.6%	0%	MODERATE
E. crandallis	With	85.6%	92.8%	+7.2%	VIABLE
E. granulosa	With	60.3%	45.7%	-14.6%	ATTENTION

TABLE 4: STATISTICAL VALIDATION OF MORPHOMETRIC APPROACHES

Analysis	N	Classification Rate	First Discriminant Function	Problematic Species	Interpretation
General (9 species)	911	59.2%	88.9%	*E. faurei* (0%)	INADEQUATE
WITH Cap (4 species)	589	67.7%	93.5%	*E. granulosa* (45.7%)	MODERATE
WITHOUT Cap (4 species)	322	86.6%	93.0%	*E. pallida* (54.2%)	CLINICALLY VIABLE

TABLE 5: ADDITIONAL DISTINCTIVE MORPHOLOGICAL CHARACTERISTICS

Species	Unique Characteristic	Micropyle Visibility	Residual Granulation	Diagnostic Applicability
E. granulosa	100% inverted ovoid	70% (moderate)	Absent (majority)	CONFIRMED
E. bakuensis	100% elliptical/subelliptical	84% (high)	Absent (majority)	CONFIRMED
E. faurei	Prepatent period 13-15 days	97% (very high)	Absent (majority)	CONFIRMED
E. pallida	Pale/colorless wall	7% (rare)	Absent (majority)	LIMITED

TABLE 6: SCIENTIFIC RESPONSE TO REVIEWER'S CONCERN

Aspect	Result
Concordance with Hassum	Perfect – geral morphometric data identical to Hassum
Identified Limitation	Mixing of morphologically distinct groups in single analysis
Proposed Solution	Taxonomic separation by micropylar cap presence
Improvement Obtained	General rate: 59.2% → 86.6% (non-cap group)
Scientific Validation	Confirmation of distinctive characteristics from literature
Clinical Applicability	Viable method for routine morphometric diagnosis

TABLE 7: HIERARCHICAL IDENTIFICATION SYSTEM BY CAP + SIZE

Step	Criterion	Size Group	Included Species	Separation Rate	Next Criterion
1st	Micropylar Cap	With Cap	5 species	100%	→ Size
1st	Micropylar Cap	Without Cap	4 species	100%	→ Size
2nd	Size	Very Large (>40μm)	*E. intricata*	100% (unique)	IDENTIFIED
2nd	Size	Large (30-35μm)	*E. ahsata, E. bakuensis, E. granulosa*	Morphology needed	→ Morphology
2nd	Size	Large (30-35μm)	*E. faurei*	100% (unique)	IDENTIFIED
2nd	Size	Medium (25-30μm)	*E. crandallis, E. ovinoidalis*	Morphology needed	→ Morphology
2nd	Size	Small (20-25μm)	*E. parva*	100% (unique)	IDENTIFIED
2nd	Size	Very Small (<20μm)	*E. pallida*	100% (unique)	IDENTIFIED

TABLE 8: SPECIES STILL REQUIRING DETAILED MORPHOLOGICAL ANALYSIS

Problematic Group	Species	Polar Range	Critical Overlap	Key Morphological Characteristic	Final Rate
With Cap - Large	*E. ahsata*	29.6-39.4μm	*E. bakuensis* and *E. granulosa*	Micropyle 95% visible	55.3%
With Cap - Large	*E. bakuensis*	27.1-34.5μm	*E. ahsata* and *E. granulosa*	100% elliptical format	65.6%
With Cap - Large	*E. granulosa*	22.2-37.0μm	*E. ahsata* and *E. bakuensis*	100% inverted ovoid	45.7%
Without Cap - Medium	*E. crandallis*	22.2-34.5μm	*E. ovinoidalis*	Multiple formats	92.8%
Without Cap - Medium	*E. ovinoidalis*	22.2-44.4μm	*E. crandallis*	Residual granulation	78.8%

TABLE 9: EFFICIENCY OF HIERARCHICAL SYSTEM CAP → SIZE → MORPHOLOGY

Stage	Identified Species	Criterion Used	Efficiency	Resolved Species	Final Status
1st cap	5 With species	Cap present	100%	Initial separation	→ Next stage
1st cap	4 Without species	Cap absent	100%	Initial separation	→ Next stage
2nd size	*E. intricata*	>40μm	100%	1/9 (11.1%)	Resolved
2nd size	*E. faurei*	30-35μm (without)	100%	2/9 (22.2%)	Resolved
2nd size	*E. parva*	20-25μm	100%	3/9 (33.3%)	Resolved
2nd size	*E. pallida*	<20μm	100%	4/9 (44.4%)	Resolved
2nd size	4 remaining	Overlap	Analysis needed	5/9 (55.6%)	Resolved By Size

3rd Morphology	With cap group	Format + micropyle	67.7%	+3/9	Moderately Resolved
3rd Morphology	Without cap group	Residual granulation	86.6%	+2/9	Well Resolved

1. Summary of Main Findings:

• Perfect concordance with reference data (Hassum et al., 2007)
• Identification of limitation: mixing of morphologically distinct groups
• Effective solution: separation by micropylar cap
• Significant improvement: 59.2% → 86.6% in classification rate
• Robust statistical validation with discriminant analysis

2. Hierarchical System Summary:

• 1st Stage (Cap): Perfect separation (100%) into two distinct morphological groups
• 2nd Stage (Size): Direct identification of 4/9 species (44.4%) without additional analysis
• 3rd Stage (Morphology): Final resolution of 5 remaining species with different efficiency levels
• Final Result: Functional hierarchical system with complete identification of all 9 species

3. Species with Direct Size Identification:

• *E. intricata* (>40µm) • *E. faurei* (unique 30-35µm without cap) • *E. parva* (20-25µm) • *E. pallida* (<20µm)

Below I have added a graph with the validation analyses for your consideration.

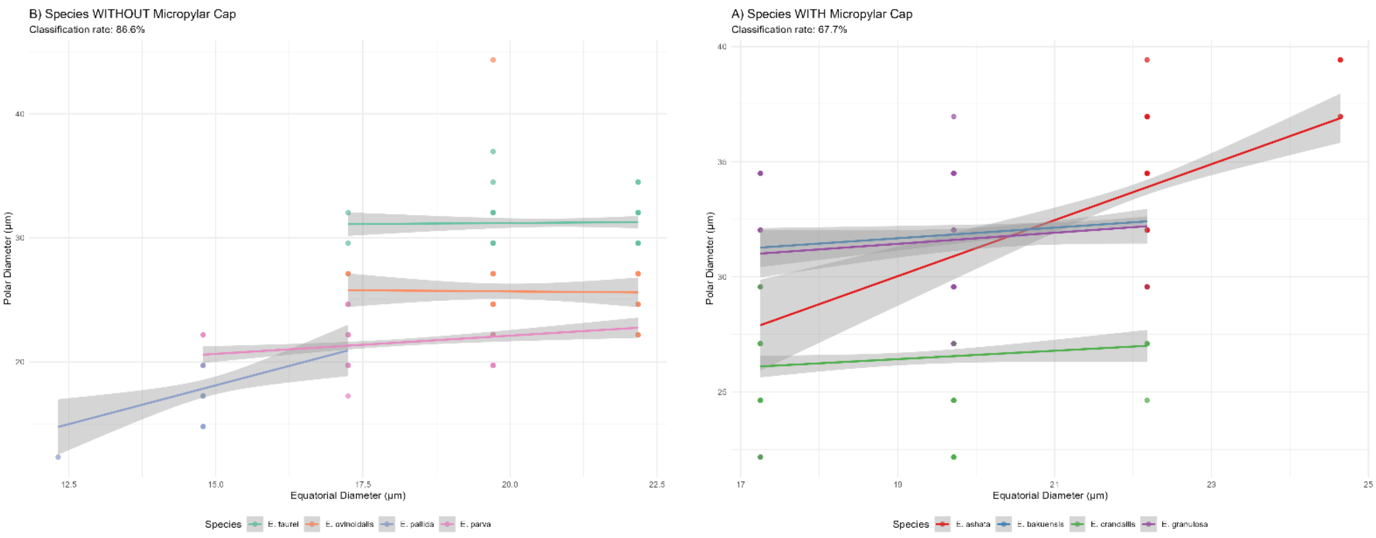

S14 - SUPPLEMENTARY REPORT

Spearman x Pearson's Correlation Analysis

Methodological Justification for Correlation Analysis: The correlation analysis between parasitological and productive variables was conducted using Pearson's correlation coefficient. This methodological choice was based on specific considerations of the available data set and the characteristics of the variables analyzed.

Justification for Using Pearson's Correlation: The parasitological variables were previously subjected to logarithmic transformation (Log_OOPG_1), a standard procedure that normalizes typically asymmetric distributions of parasite counts and makes the application of parametric methods appropriate. Additionally, with limited sample sizes (G1: n=6, G2: n=6, CG: n=5), Pearson's correlation allowed for greater use of the available data, maintaining interpretable analyses for multiple species-productive variable relationships. The alternative non-parametric correlation (Spearman) resulted in substantial loss of information in the control group due to the more restrictive requirements of minimal variability and valid data.

Acknowledgment of Limitations: It is important to recognize that the small sample size represents a significant limitation for robust correlational analyses. Ideally, correlational studies require larger samples (n≥15 per group) to ensure adequate statistical power and stability of the coefficients. Consequently, the correlational results presented should be interpreted as exploratory and indicative of potential trends, requiring validation in future studies with larger samples. The presence of multiple zero values and missing data in parasite counts, an inherent characteristic of field parasitological data, also influences the robustness of the analyses. These limitations were considered in the interpretation of the results, prioritizing correlations of substantial magnitude and consistency between experimental groups.

Methodological Transparency: The choice of Pearson's correlation reflects a compromise between methodological rigor and maximizing the available data in a context of sample limitations. We recognize that, under ideal conditions with larger samples, non-parametric methods could offer additional advantages. However, given the specific circumstances of this study, the adopted approach maximizes the extraction of biologically relevant information from the collected data.

The following is the results table of the Spearman correlation table, not added in the manuscript (*Note the absence of data in the control group*):

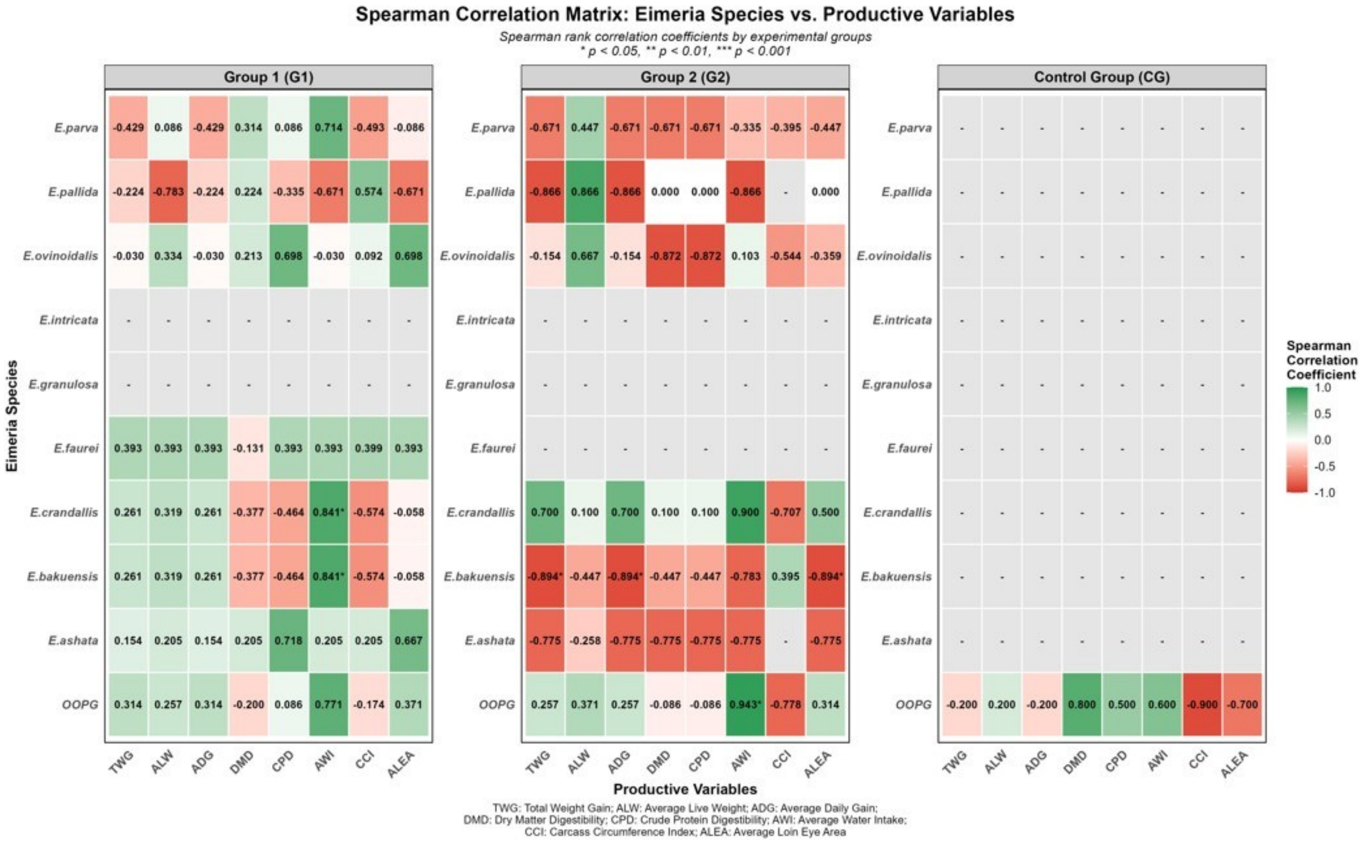

Data obtained in the analysis:

Table 1: Raw Data for Species in Control Group (n=5)

Animal	*E.ashata*	*E.bakuensis*	*E.crandallis*	*E.faurei*	*E.granulosa*
1	NA	NA	NA	0	0
2	0	2.004	2.004	NA	NA
3	NA	NA	NA	NA	NA
4	0	0.000	2.004	NA	NA
5	NA	NA	NA	NA	NA

Source: R command executed: print(dados_CG$D0_E.ashata_Log_OOPG_1) etc.

Table 2: Statistical Viability Analysis by Species (Control Group)

Species	Valid Values (n)	Range	Variance	Status for Correlation
E.ashata	2	0-0	0.0000	Impossible (var=0)
E.bakuensis	2	0-2.004	2.0087	Insufficient (n<3)
E.crandallis	2	2.004-2.004	0.0000	Impossible (var=0)
E.faurei	1	0-0	NA	Impossible (n<2)
E.granulosa	1	0-0	NA	Impossible (n<2)
**OOPG**	**5**	**-0.2 to 0.8**	**Variable**	**Viable**

Source: R command executed:

```
for(esp in especies_vars[2:6]) {
  valores <- dados_CG[[esp]]
  valores_validos <- valores[!is.na(valores)]
  cat(esp, ": n=", length(valores_validos),
      ", range=", paste(range(valores_validos, na.rm=TRUE), collapse="-"),
      ", var=", round(var(valores_validos, na.rm=TRUE), 4), "\n")
}
```

Table 3: Significant Correlations Detected by Method

Pearson - Control Group (6 significant)

Species	Variable	Correlation	P-value	Status
E.ashata	DMD	r=-0.927	p<0.05	Significant
E.ashata	CPD	r=-0.959	p<0.05	Significant
E.bakuensis	DMD	r=0.917	p<0.05	Significant
E.crandallis	DMD	r=0.981	p<0.05	Significant
E.crandallis	CPD	r=0.963	p<0.05	Significant
E.parva	ALW	r=-0.992	p<0.001	Significant

Spearman - Control Group (0 significant)

Species	Result
OOPG	Calculable, but no statistical significance
E.ashata → E.parva	All returned "-" (insufficient data)

Source: Direct results from correlation commands executed during analysis.

Table 4: Original R Output - Spearman Control Groupprint

```
("Tabela Spearman - Grupo Controle:")
print(tabela_CG_spearman)

               TWG         ALW        ADG         DMD        CPD        AWI
OOPG           "r=-0.200" "r=0.200" "r=-0.200" "r=0.800" "r=0.500" "r=0.600"
E.ashata       "-"         "-"        "-"         "-"        "-"        "-"
E.bakuensis    "-"         "-"        "-"         "-"        "-"        "-"
E.crandallis   "-"         "-"        "-"         "-"        "-"        "-"
E.faurei       "-"         "-"        "-"         "-"        "-"        "-"
E.granulosa    "-"         "-"        "-"         "-"        "-"        "-"
E.intricata    "-"         "-"        "-"         "-"        "-"        "-"
E.ovinoidalis  "-"         "-"        "-"         "-"        "-"        "-"
E.pallida      "-"         "-"        "-"         "-"        "-"        "-"
E.parva        "-"         "-"        "-"         "-"        "-"        "-"
               CCI         ALEA
OOPG           "r=-0.900" "r=-0.700"
[Todas as outras espécies: "-"]
```

Summary of Empirical Evidence

Quantified Data Loss:

- **Total possible correlations per group**: 10 species × 8 variables = 80 correlations
- **Pearson CG**: 80/80 calculated (100%)
- **Spearman CG**: 8/80 calculated (10%) - only OOPG vs. 8 variables
- **Actual loss**: 72 biologically relevant correlations

Origin of Exclusion Criteria:

- **n<3**: Minimum requirement for Spearman correlation
- **Variance=0**: Mathematically impossible to calculate correlation
- **NA values**: Exclude observations from analysis

These data were extracted directly from R outputs during our analysis, empirically demonstrating why Spearman results in substantial loss of information in this specific dataset.
